# Gender Felt Pressure, Affective Domains, and Mental Health Outcomes among Transgender and Gender Diverse (TGD) Children and Adolescents: A Systematic Review with Developmental and Clinical Implications

**DOI:** 10.3390/ijerph20010785

**Published:** 2022-12-31

**Authors:** Selene Mezzalira, Cristiano Scandurra, Fabrizio Mezza, Marina Miscioscia, Marco Innamorati, Vincenzo Bochicchio

**Affiliations:** 1 Department of Humanities, University of Calabria, 87036 Rende, Italy; 2 Department of Neuroscience, Reproductive Sciences, and Dentistry, University of Naples Federico II, 80131 Naples, Italy; 3 SInAPSi Center, University of Naples Federico II, 80133 Naples, Italy; 4 Department of Developmental Psychology and Socialization, University of Padua, 35131 Padua, Italy; 5 Department of History, Cultural Heritage, Education, and Society, University of Rome Tor Vergata, 00133 Rome, Italy

**Keywords:** transgender, youth, mental health, gender pressure, minority stress

## Abstract

Although capable of mobilizing significant resilience factors to face stigma and discrimination, transgender and gender diverse (TGD) children and adolescents tend to suffer from more adverse mental health outcomes compared to their cisgender counterparts. The minority stressors that this population faces are mainly due to the gender-based pressure to conform to their assigned gender. This systematic review was aimed at assessing the potential mental health issues that affect the TGD population. The literature search was conducted in three databases; namely, Scopus, PubMed, and Web of Science, based on the PRISMA guidelines. The 33 articles included in the systematic review pointed out how TGD children and adolescents experience high levels of anxiety and depression, as well as other emotional and behavioral problems, such as eating disorders and substance use. Resilience factors have been also pointed out, which aid this population in facing these negative mental health outcomes. The literature review highlighted that, on the one hand, TGD individuals appear to exhibit high levels of resilience; nonetheless, health disparities exist for TGD individuals compared with the general population, which are mainly attributable to the societal gender pressure to conform to their assigned gender. Considerations for research and clinical practice are provided.

## 1. Introduction

### 1.1. Definitional Issues

Even though the term “identity” stems from the Latin “identitas,” meaning “the same”, individuals might have various identity features based on gender, ethnicity, religion, nationality, and so forth [1]. Gender identity trajectories are complex processes, substantially influenced by psychological (e.g., cognitive, emotional), and social (e.g., interpersonal, familiar, community-related) factors [2]. Gender identity is not to be confused with gender expression and sexual orientation. Whereas gender identity refers to the individual experience of one’s gender, gender expression refers to how a person publicly expresses or exhibits their gender. Sexual orientation refers instead to who the person is attracted to and wishes to have relationships with (e.g., heterosexual, gay, lesbian, bisexual, asexual, etc.). In the field of transgender studies, “affirmed gender” is used to denote the gender that an individual asserts themselves to be.

Transgender and gender diverse (TGD), gender variant, gender queer, and gender nonconforming are all umbrella terms, which describe those individuals whose gender identity, expression, role, or behavior do not conform to the social norms based on their socially assigned gender [3,4,5]. Whereas “gender dysphoria” refers to the perceived distress of individuals whose gender identity differs from that assigned at birth, and represents a psychiatric diagnosis for the American Psychiatric Association (DSM-5) [6,7], TGD encompasses various discrepancies between the gender assigned at birth and the individual’s experienced gender identity, role, or expression [8]. The prevalence of TGD among young individuals seems to be more common among male than female adolescents; specifically, TGD appears as more prevalent among homosexual, bisexual, and “not sure” adolescents than their heterosexual peers, and to be inversely proportional to school grade level as well [9].

### 1.2. Routes to Gender Affirmation

An array of procedures is available for TGD youth to affirm their perceived gender, also based on the developmental stage of the target person. First, “social transition” refers to a nonmedical procedure whereby the young person adopts a gender identity (e.g., by changing name and pronouns), gender expressions (e.g., by changing hairstyle, clothing, etc.), and gender roles that match their asserted gender [10,11]. Social transition can increase the feeling of one’s alignment with the affirmed gender [12]. Social transition includes the so-called gender identity “appearance congruence” (AC) (i.e., the alignment that the person perceives with their gender identity when compared with the physical and/or anatomical appearance), which seems to be inversely correlated to TGD individuals’ mental health [13]. In fact, in addition to enhancing AC, the social transition of prepubescent TGD children is associated with levels of depression and anxiety comparable with those of their cisgender peers [14]. Second, hormone treatment (via the so-called “hormone blockers”) leads to pubertal suppression, and is often prescribed to transgender adolescents to pause their puberty [15]. Finally, medical surgery procedures directly intervene on specific body parts in order to modify or remove them, especially with regard to sex organs [16]. Overall, research has shown that being in the earlier stages of gender transition or affirmation can be a stressful condition that negatively impacts the person’s mental health [17]. 

### 1.3. TGD Youth’s Mental Health and Emotional or Affective Functioning

TGD individuals tend to suffer more from more adverse mental health outcomes compared with their cisgender counterparts [18,19,20], but the causes of such health disparities are still widely unknown [12]. Specifically, TGD adolescents tend to experience higher rates of depression than their cisgender peers, and to engage in more self-harm behaviors [21]. In particular, more than half of transgender adolescents have a diagnosis of depression; half of these also experience suicidal ideation and almost one third attempt suicide [22]. Anxiety and depression are associated with emotional, affect-related, and cognitive factors (e.g., negative future expectations; [23,24]). In addition to depression and anxiety, young TGD individuals also tend to experience gender dysphoria due to their TGD; in turn, gender dysphoria has been deemed to be associated with the high prevalence of mental illness in the TGD population [25]. TGD individuals tend to also be at a higher risk of substance use compared with the general population [26].

### 1.4. TGD Youth, Gender Pressure, and Minority Stress

The minority stress theory (MST) posits that individuals belonging to social minority groups face higher levels of sociocultural stressors compared with non-minority groups, and that such stressors negatively affect their mental health outcomes [27,28,29,30]. Within the MST framework [28,29], minority stressors lie on a distal-proximal axis. They are said to be “distal” when they are caused by external factors that marginalize the person or represent a threat to their safety and security (e.g., discrimination, prejudice, stereotype, harassment, verbal or physical assault, hate crime, microaggressions, and denial of access to services or opportunities). Instead, they are said to be “proximal” when referring to subjective beliefs, feelings, and thoughts of a person involved in an environment perceived as unsafe, stigmatizing, and oppressive (e.g., expectations of rejection, internalized stigma, and concealment to gendered social norms to avoid prejudice). On the positive side, however, specific resilience factors been shown to possibly buffer the negative effects of minority stressors on mental health outcomes [31]. Group-level resilience factors include social support and the feeling of connectedness with other members of one’s community [32], whereas individual-level resilience factors refer to personal characteristics (e.g., agency, self-worth, and pride) that help the person face the challenges posed by the stress they face due to their belonging to a minority group [33]. The MST has been further applied to understand the increased risk of mental health outcomes in TGD individuals [34,35,36,37,38,39,40]. Within the Gender Minority Stress and Resilience theory, which was developed as an extension of the MST to include the specific types of minority stress faced by TGD individuals and the corresponding resilience factors [41], gender-specific stressors refer, for instance, to non-affirmation, which consists of ignoring or invalidating one’s gender identity [42], and to internalized transnegativity, resulting from social stigma to TGD identities [43,44,45]. In contrast, TGD individuals’ resilience factors include self-definition (of one’s gender identity), and deciding to transition, whether through a medical or social procedure [46]. 

### 1.5. The Present Work

Reviews exist that address young TGD individuals [47,48,49,50]. These reviews either specifically focus on school-related variables [47], deal with attachment and mentalization [48], or, are intended to analyze the relationship between religion, spirituality and mental health [49]. A review also exists as to the mental health of TGD youth based on the minority stress framework [50], yet no mention is made about the construct of gender pressure or “felt pressure” [51], which is extremely important in understanding and explaining the type of stress experienced by this population. Our systematic review is aimed at investigating the mental health of TGD youth (i.e., children and adolescents up to 18 years of age) from a psychodynamic perspective, analyzing their affective domains, such as emotions, affects, mood, and overall mental health problems, taking into account the conceptualization of the gendered felt pressure that society tends to impose on this population.

## 2. Methodology

### 2.1. Search Strategy

We conducted a systematic review aimed at investigating the mental health of TGD children and adolescents, and at exploring the related variables that can significantly impact a TGD youth’s quality of life. As to the systematic review, we followed the Preferred Reporting Items for Systematic Reviews and Meta-Analyses (PRISMA) guidelines [52]. A systematic search was conducted in three databases: Scopus, PubMed, and Web of Science. The search strategy was based on the use of Boolean operators to combine terms related to TGD, youth, and mental health. The search terms used to identify eligible articles comprised: [(transgender OR gender diverse OR gender varian* OR gender nonconform*) AND (child* OR adolesc* OR youth OR young*) AND (mental health OR mental problem* OR psychopatholog* OR mental disorder*)]. Searches for eligible articles in the Scopus, PubMed, and Web of Science databases were conducted from 1 August 2022 to 19 September 2022. 

### 2.2. Eligibility Criteria

The inclusion criterion, which guided the data extraction process, consisted of selecting articles that specifically addressed mental health-related variables in TGD youth (i.e., children and adolescents). To be included, studies had to meet all the following criteria: (1) being published in peer-reviewed journals; (2) having a sample comprised of TGD young individuals (up to 18 years of age); (3) including original data; (4) including quantitative results; (5) containing at least one variable related to the mental health of TGD children and/or adolescents. Exclusion criteria were applied for non-English texts, non-indexed and non-peer-reviewed records, studies lacking quantitative data, and grey literature (i.e., books, book chapters, theoretical articles, commentaries, editorials, etc.).

### 2.3. Selection Process

**Figure 1:**
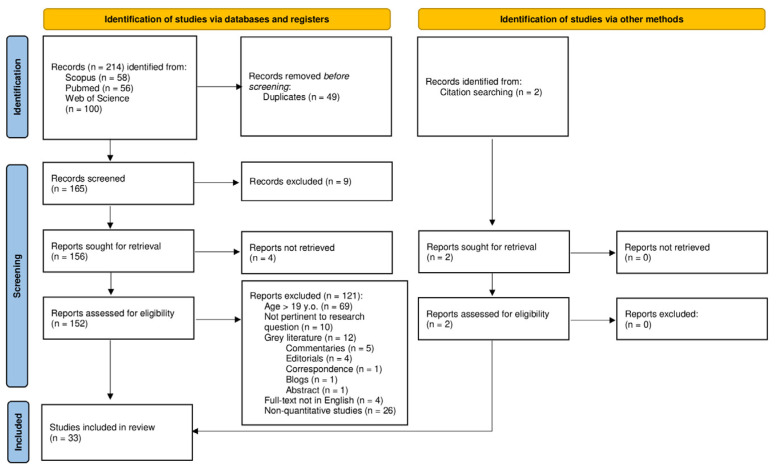
PRISMA 2020 flow diagram. From: Page MJ, McKenzie JE, Bossuyt PM, Boutron I, Hoffmann TC, Mulrow CD, et al. The PRISMA 2020 statement: an updated guideline for reporting systematic reviews. BMJ 2021;372:n71. doi: 10.1136/bmj.n71 [52]. For more information, visit: http://www.prisma-statement.org/ (accessed on 23 November 2022).

The initial search identified a total of 214 publications. After collecting the records and removing the duplicates, we applied the eligibility criteria. A total of 165 records were screened by SM and CS, assessing titles and abstracts according to the inclusion criteria. Disagreements between the first three authors were settled through the involvement of two other authors, namely, FM and MM. In total, 9 records were excluded in the screening process, which resulted in the retrieval of 156 records. The full-text of these records were obtained and reviewed by SM and FM, and any discrepancy over eligibility determination was resolved through involvement of MM and MI. Among the 156 full-text articles sought for retrieval, 4 full-texts were unavailable, resulting in 152 full-texts assessed for eligibility. Of these, 121 records did not meet the inclusion criteria, and were thus excluded from the systematic search. Specifically, 69 records were excluded due to the presence of a sample that did not match the age constraints (<18 years of age), 10 were not pertinent to the research question, 4 full-texts were not in English, 26 were non-quantitative studies, and 12 involved grey literature. Of the latter, five were commentaries, four were editorials, one was a correspondence, one a blog, and one an abstract. Given that all databases used for our search allowed for the emergence of records containing the search terms in the title, abstract, and keywords, according to the PRISMA guidelines, in-text citations of the selected articles were then further inspected to identify additional records. As a result, in-text relevant citations, if peer-reviewed and indexed, were also considered. The systematic search eventually led to the inclusion of 33 articles. The details of this procedure are illustrated in Figure 1.

### 2.4. Data Extraction Process

Data were extracted from each full-text paper, which included: the author(s)’ last name, year of publication, information on the country of study performance, study design, sample characteristics (sample size and age), types of respondents, and mental health outcome measures. Data extraction was cross-checked by all co-authors.

### 2.5. Quality Assessment

The National Institutes of Health’s Quality Assessment Tool for Observational Cohort and Cross-Sectional Studies was used to rate the quality of the studies included in the review. This tool is composed of 14 items that assess various factors associated with the internal validity of the study, including the clarity of the research question and methods, representativeness of the study sample and selection biases, sample size justification, appropriateness of the study measures, and so forth. Each study included in the current review was scored for each of the 14 domains as: yes, no, cannot determine, not applicable, not reported. Based on these scores, we obtained an overall rating determining each study’s quality as poor, fair or good. Quality assessment of the studies was completed independently by SM and CS. Cohen’s kappa was used to calculate the agreement between evaluators and yielded a score of (*κ* = 0.85), indicating strong agreement. Any discrepancies were solved by discussion between the assessors and two additional reviewers (FM and MM).

## 3. Results

### 3.1. Mental Health Outcomes

All details of the included articles have been outlined in Table 1.

More than one third (n = 13) of the 33 included articles addressed internalized psychopathology such as depression and/or anxiety [12,14,53,54,55,57,58,60,64,66,68,69,71,76]. The included contributions also addressed other mental health outcomes and/or problematic behavior such as attention deficit and/or hyperactivity disorder (ADHD) and conduct disorder (CD) [53], autism spectrum disorder (ASD) [53,63,66,73], eating disorders [53,66], trauma, harassment, bullying, and victimization [55,57,58,66,69,76,79], adverse childhood experiences (ACEs) [56,71], substance use [9,26,58,71,80], and sex trading [67]. A few of the included articles (n = 8) also involved resilience factors [12,14,54,57,58,60,61,76].

### 3.2. Types of Respondents

Respondents were either TGD youth (n = 14), caregivers (n = 8), both TGD youth and caregivers (n = 5, one of which also included cisgender siblings), both TGD and their cisgender peers (n = 3), medical doctors (n = 2), and one study had adults retrospectively recall the age when they socially transitioned.

### 3.3. Depression and Anxiety

Overall, the findings outlined in the included articles point to the fact that various mental health issues, especially depression and anxiety, are more common in TGD youth than in the general population. Nahata and colleagues [66] found that more than 90% of their sample of TGD adolescents referred to pediatric endocrinology had a diagnosis of either depression, anxiety, post-traumatic stress disorder (PTSD), eating disorders, ASD, or bipolar disorder. Moyer and colleagues [64] also found high rates of depression, anxiety, and suicidal ideation among TGD adolescents. Similarly, Parodi and colleagues [69] found that, among TGD adolescents, anxiety was the most common mental health concern, followed by depression, non-suicidal self-injury, and PTSD, respectively. Comparing rates of depressive symptoms among TGD and cisgender Thai adolescents, Cheung and colleagues [55] found that the highest burden of depression affected gender nonconforming girls, followed by gender nonconforming boys, gender-conforming girls, and gender-conforming boys, respectively. Becerra-Culqui and colleagues [53] found that the most common diagnoses among TGD children were anxiety disorders and ADHD, which were reported at higher levels than in their cisgender peers. Consistent with these findings, Russell and colleagues [72] also found that, if compared with non-TGD children, TGD youth reported higher levels of mental health issues on various dimensions, including depression, anxiety, somatic concerns, conduct problems, and suicidality. In Stewart and colleagues’ [74] study, TGD youth reported significantly higher levels of anxiety, depression, social disengagement, positive symptoms, high levels of risk for suicide and/or self-harm, and were more likely to experience emotional abuse, past suicide attempts, and weaker and less supportive family relationships than cisgender (female and male) young individuals. In Katz-Wise and colleagues’ [60] sample of TGD youth, high levels of reported suicidal thoughts were found, along with suicidal plans, suicidal ideation, and suicidal attempts, respectively; also, almost half of the TGD participants reported lifetime self-harm, and more than half of the sample reported clinically significant depressive symptomatology. Lowry and colleagues [9] found that feelings of sadness and hopelessness seemed to increase with TGD among both male and female adolescents (albeit not linearly); also, seriously considering suicidal attempt, as well as having a suicide plan, was found to increase along with TGD among both males and females. As to the potential precipitating factors of poor mental health outcomes, Chodzen and colleagues [12] found that TGD adolescents high in internalized transphobia (i.e., a “proximal” stressor in the gender minority stress framework; [28]) were more likely to be affected by Major Depressive Disorder (MDD) and Generalized Anxiety Disorder (GAD) than their cisgender peers. In line with these findings, Wang and colleagues [79] showed that, in comparison with their cisgender peers, TGD adolescents reported higher levels of depression and anxiety symptomatology, sleep problems, suicidal ideation, and self-harm thoughts and behaviors, and that this population also had higher odds of being bullied at school. Aiming at exploring the differences in the subjective reports of mental health and psychosocial functioning in TGD adolescents completing the Child Behavior Checklist (CBCL) [81] and the Youth Self Report (YSR) [82], Kuper and colleagues [62] found that almost half of all participants scored in the clinically significant range on Total Problems, Internalizing Problems, and Total Competency scales, also suggesting that the relatively high rates of the first two scales were driven by high levels in Depressive Problems, Anxiety Problems, Obsessive Compulsive Problems, and Post-Traumatic Stress Problems. As opposed to Externalizing Problems and Competency-related Problems, where no gender differences were found, transgender males reported significantly higher scores than transgender females in all internalizing scales. Regarding the nonbinary population—which has been shown to suffer from higher levels of mental health problems compared to binary individuals [83]—Moyer and colleagues [64] found that these individuals tended to endorse more suicidal ideation than their TGD (male and female) counterparts. More specifically, Parodi and colleagues [69] found that nonbinary assigned female at birth (AFAB) adolescents reported higher levels of depressive distress if compared with transgender females. In one study including a sample of ethnic minorities, Vance and colleagues [76] found comparable levels of mental health outcomes (i.e., depressive symptomatology and suicidal ideation), victimization, and harassment between Black and Latinx transgender youth and their White transgender counterparts, even though the former reported lower levels of school connectedness; compared with their Black and Latinx cisgender peers, however, Black and Latinx transgender individuals reported higher levels of depressive symptoms and suicidality, higher odds of being harassed, higher levels of victimization, and lower levels of psychosocial protective factors, such as school connectedness. However, these findings seem not to be universally suggested in all studies. For instance, Durwood and colleagues [14], found that, consistent with their parents’ reports, TGD children reported higher levels of anxiety but not higher levels of depression and self-worth than their matched-control or sibling peers. In line with these results, Olson and colleagues [68], albeit finding higher levels of anxiety among TGD prepubescent children who had already socially transitioned if compared with their cisgender counterparts, also found that levels of depressive symptomatology were not significantly different between the two groups.

### 3.4. Emotional and Behavioral Problems

Comparing a group of TGD adolescents with a group of cisgender youth, Perl and colleagues [70] found that, consistent with previous research [84], the COVID-19 pandemic had negative emotions as a consequence for all groups. However, if compared with their cisgender peers, TGD adolescents reported more negative emotions, more media use, and less sport activity than their cisgender counterparts. Negative emotions, which increased during COVID-19, appeared to decrease at follow-up 3 months later. Cognitive reappraisal was also stronger than suppression for cisgender individuals, but did not differ in TGD youth [70]. VanderLaan and colleagues [77] found that higher levels of separation anxiety in assigned male at birth (AMAB) children referred to a gender dysphoria clinic were significantly associated with TGD, increased behavioral and emotional problems, and poor peer relationships. Identifying children whose score levels on the GIQC were comparable with those typically exhibited by children who are clinic-referred for gender dysphoria, Van der Miesen and colleagues [78] found that, overall, rates of clinical-range scores in the CBCL among TGD children were consistent with those reported by gender dysphoria-referred children. Reidy and colleagues [71] showed that gender role discrepant boys who were distressed about this issue were more likely to engage in risky behavior and to report poorer mental health. However, those who were not distressed by their gender role nonconformity were less likely to engage in risky behaviors. Nic Rider and colleagues [67] found that, among 9th and 11th graders of their sample, almost 6% reported trading sex, and that TGD adolescents who traded sex had higher levels of mental health concerns than their peers; also, more than 75% of TGD individuals trading sex reported a lifetime suicide attempt, even though rates were also high among TGD youth who never traded sex and among cisgender students who traded sex. Reisner and colleagues [26] found that TGD youth were more likely to have used alcohol, cigarettes, marijuana, and nonmarijuana illicit drugs in the past 12 months compared to cisgender boys, and to regularly make use of marijuana and illicit drugs. This population also reported significantly higher numbers of past-12-month bullying and harassment events than cisgender youth. Eisenberg and colleagues [58] also found that TGD youth reported significantly higher levels of involvement in health risk behaviors (i.e., substance use, sexual behavior, emotional distress, and bullying victimization). Examining the associations between the recalled age of social transition and adult mental health outcomes, Turban and colleagues [75] found that social transition during childhood was associated with lower odds of lifetime marijuana use when compared with those who socially transitioned during adulthood, and that harassment exposure was more common among those who socially transitioned during childhood. In addition, there was no difference between childhood and adolescent social transition on any mental health outcomes examined (e.g., psychological distress and substance use). Eisenberg and colleagues [58] found that AMAB individuals tended to engage in binge drinking, use of marijuana, high-risk sexual behaviors, and physical bullying more than AFAB individuals. Watson and colleagues [80] found that TGD youth reported lower cigarette use when living in states with nondiscrimination and anti-bullying laws; however, TGD individuals reported more alcohol use and binge drinking in states with such laws, perhaps due to increased socialization habits. Finally, Lowry and colleagues [9] found that TGD was significantly associated with substance use only among male adolescents and not among females.

### 3.5. Trauma and Victimization

ACEs are specific traumatic events that have been found to have overall negative long-term consequences for the individuals’ mental and physical health [85]; for instance, heightening the levels of psychological distress, depression, anxiety, and suicidal ideation [86,87]. ACEs tend to be highly co-occurring among TGD youth [88]. Reidy and colleagues [71] found that traumatic experience, ACEs, and neighborhood disorganization had all negative effects on this populations’ psychosocial maladjustment, and that gender role discrepancy (GRD) was positively associated with higher levels of traumatic symptomatology, which, in turn, was positively associated with masculine discrepancy stress (MDS). Based on research showing that the prevalence of ACEs among lesbian, gay, bisexual, or transgender (LGBT) adults is much higher than in heterosexual individuals [89], Craig and colleagues [56] found that TGD youth are significantly more likely to report higher ACE scores than cisgender individuals. In Clark and colleagues’ [90] study, TGD high school students were found to be at increased risk of violence, mistreatment, and poor safety compared with their cisgender peers. Finally, Watson and colleagues [80] found that TGD youth were less likely to experience bullying if they lived in states with nondiscrimination, anti-bullying laws, or “conversion therapy” laws, and that, conversely, they were more likely to experience bullying if they lived in states that had anti-LGBT laws; however, in these latter states, alcohol use and abuse was also more common.

### 3.6. Family-Related Factors

Munroe and colleagues [65] found that, among TGD children, experiencing difficulties in either peer or family relationships was associated with internalizing symptomatology, whereas poor peer (but not family) functioning was associated with externalizing symptoms. Aiming at examining the relationships between parent-related variables and TGD children’s psychological functioning, Kolbuck and colleagues [61] found that parenting stress significantly predicted higher levels of symptomatology related to a variety of mental health diagnoses (i.e., ADHD, oppositional defiant disorder, CD, GAD, MDD, dysthymia, and social anxiety disorder). Interestingly, however, Katz-Wise and colleagues [60] found that TGD adolescents reported high levels of psychological distress even when they belonged to family systems that were supportive of them seeking medical care and/or taking part in research projects. In this regard, Hill and colleagues [59] found that pathological tendencies of TGD children and adolescents could not be predicted by either parents’ attitudes toward them being TGD, or the degree of TGD they exhibited.

### 3.7. TGD and ASD

Mahfouda and colleagues [63] found that TGD children diagnosed with ASD were not only overrepresented in their TGD sample, but also reported higher levels of internalizing behavior on the YSR scale. TGD youth diagnosed with ASD also tended to experience reduced quality of life in various domains (i.e., physical health, social and emotional well-being, and school functioning) compared to other TGD participants. Shumer and colleagues [73] also found a significant association between higher autistic traits in children or their mothers and higher degrees of TGD in children.

### 3.8. External Factors

Based on the recognition of the role that puberty blockers play in allowing TGD youth to experience gender continuity without being forced to experience somatic changes associated with the gender they experience as “wrong”, Nahata and colleagues [66] found that TGD adolescents, who were denied insurance coverage for puberty blockers and were not able to personally cover the costs (thus remaining untreated), were at a particularly high risk for suicide attempts, suicidal ideation, and self-harm. In addition to experiencing high levels of mental health problems, Clark and colleagues [90] found that TGD youth had been unable to access health care when they needed it. On a vast sample of adults, Turban and colleagues [75] retrospectively investigated various mental health outcomes related to the age (specifically: 3–9 years old, 10–17 years old, and older than 18) when participants recalled starting to live full-time in a gender different to that assigned at birth. The authors found no association between social transition during childhood and negative mental health outcomes when compared with transition during adulthood. For instance, transitioning during childhood was associated with lower odds of lifetime marijuana use when compared with adult social transition.

### 3.9. Resilience Factors

Investigating the impact that various minority stress and resilience factors have on the likelihood that TGD adolescents meet the diagnostic criteria for MDD and GAD, Chodzen and colleagues [12] found that TGD adolescents who exhibited high levels of gender identity AC were less likely to be affected by MDD than those who had lower levels of AC. Therefore, gender identity AC (which includes reversible, social gender transition) seems to be a protective factor against MDD, which can, in turn, decrease through gender affirming medical interventions, such as hormone blockers and gender-affirming hormones [12]). In turn, Durwood and colleagues [57] found that parents who reported higher levels of family-, peer-, and school-based support for their TGD children’s gender identity also reported fewer internalizing symptoms; also, peer- and school-related support moderated the association between gender-related victimization and internalizing symptoms, as reported by parents. Furthermore, Katz-Wise and colleagues [60] found that better family communication and greater family satisfaction were associated with fewer mental health outcomes (i.e., less self-harm, fewer depression- and anxiety-related symptomatologies), and with greater self-esteem and resiliency among TGD adolescents. In spite of the significance of protective factors when facing minority stressors, however, Eisenberg and colleagues [58] found that several of such factors (i.e., family connectedness, teacher-student relationship, and a feeling of safety in the community) were significantly lower among TGD youth than in their cisgender peers. In addition, AMAB persons appeared to have higher levels of protective factors than AFAM, except for the feeling of safety in the community. Overall, therefore, TGD youth tends to engage more in risk behaviors and to experience poorer protective factors compared with their cisgender counterparts [58]. Finally, one study [54] included a specific training to enhance mental health and resilience factors among TGD youth. Bluth and colleagues [54] applied the Mindful Self-Compassion for Teens (MSC-T) to transgender adolescents, formerly called Making Friends with Yourself [91], which had been previously found to decrease negative affect, anxiety, depression, and stress, and to increase resilience among non-transgender adolescents [92,93]. Bluth and colleagues [54] found that, except for thwarted belongingness, all other psychological outcomes (i.e., self-compassion, depression, perceived burdensomeness, anxiety, mindfulness, resilience, and life satisfaction) significantly improved from pre- to post-intervention with the MSC-T. As a result, the authors concluded that enhancing self-compassion can aid transgender adolescents in coping with the mental health concerns they are forced to face.

### 3.10. Quality Assessment

Quality ratings indicated that the overall methodological quality of papers included in the current review was good, with the majority of the studies (n = 28) being of good quality and five of fair quality. The main area of weakness is inherently attributable to the cross-sectional nature of the included studies—which means that causality cannot be determined—and regards the sample size justification of the studies, which is rarely reported. See Table 2 for more details.

## 4. Discussion

### 4.1. Mental Health Problems among TGD Individuals: The High Prevalence of Depression and Anxiety

The vast majority of the articles included in this systematic review point to the high prevalence of often severe mental health problems among TGD youth. Depression and/or anxiety are the main mental health concerns addressed in the current literature, variously followed by other emotional and/or behavioral problems such as ADHD and CD, eating disorders, substance use, sex trading, and even neurodevelopmental disorders such as ASD. In addition, precipitating factors have been addressed (e.g., trauma, harassment, bullying, and victimization) along with protective factors (e.g., family-, peer-, and community-based support). These findings are supported by the scientific research, as attested by other reviews of the literature [47,48,49,50].

However, as opposed to previous reviews [47,48,49], which specifically focused on either reflective functioning and mentalization, religion and spirituality, or school-related factors, this systematic review not only included studies whose sample comprised both children and adolescents, but also focused on the associations between mental health, emotional and behavioral functioning, and the overall environment (e.g., family, community, and peer relationships) in which TGD children and adolescents live. A review has also been written on the protective and precipitating factors for TGD youth’s mental health based on the minority stress framework [50], yet no mention is made as to the gender-based pressures this population has to face. 

### 4.2. Gender Felt Pressure and TGD Transition

Gender conformity pressure, often referred to as “felt pressure”, refers to the experience of personal and interpersonal pressure to exhibit types of behavior that are conforming to one’s socially assigned gender [51]. The developmental challenges that TGD youth tend to experience often result from these stereotypical gender-based pressures. Indeed, considering these types of pressure is thus all the more significant, since they appear to be most common during childhood and early adolescence [94,95]. Felt pressure is negatively associated with the person’s psycho-social adjustment (e.g., low self-esteem and peer rejection [96]), along with various mental health problems [97], and different types of social interactions [98]. This suggests that clinicians must pay great attention to personal and interpersonal variables in addressing felt pressure [99], recognizing the importance of context and identity in facing this type of gender-based pressure [100].

Egan and Perry [51] argued that gender identity is a multidimensional construct. Given that self-perceived gender typicality and felt pressure for sex typing are differently associated with psychosocial adjustment, gender typicality is not to be taken as necessarily leading to poor well-being. Indeed, according to Egan and Perry [51], gender typicality is not harmful, but rather felt pressure for gender conformity. Research has shown that self-perceived TGD is related to questioning felt compatibility with the same-sex peer group among early adolescents [96]. Accordingly, the association between self-perceived TGD and felt gender typicality seems to partly account for the psychological distress that stems from perceiving oneself as having violated a gender standard. A possible way through which one’s perceived gender nonconformity impacts well-being is, thus, by undermining gender compatibility [96]. Pressure to conform to gender-based norms has been found to be associated with a perceived similarity to one’s own-gender when it is accompanied by the lack of feeling of similarity to the other-gender [101]. Ultimately, the emotional and behavioral problems reported by TGD youth should be viewed in light of socially-based prejudice, discrimination, and victimization [102]. Indeed, a link seems to exist between TGD, homophobic bullying, and psychological distress [103]. Therefore, TGD youth faces a higher risk of poor mental health and psychological distress especially due to the social discrimination and harassment they often experience [104]. Overall, these findings might suggest that it is not TGD nor social transition per se that is harmful, but unaccepting and stigmatizing environments [75].

### 4.3. Developmental Challenges of Children and Adolescents

The developmental challenges of TGD children and TGD adolescents are quite different. For instance, AC has been supposed to generally decrease the levels of mental health outcomes, such as MDD and GAD, in this population [12]. However, whereas in children AC can consist of a pure social transition, which has been found to decrease health disparities between this population and non-TGD individuals [105], among TGD adolescents, this can also include changes in anatomical characteristics through gender-affirming medical interventions such as puberty suppression or hormone therapy, which appear to be beneficial for the TGD person’s quality of life [106].

### 4.4. Minority Stress and Trauma

Within the minority stress framework [28,41], a “proximal stressor” such as IT has been shown to negatively impact the mental health of TGD youth [12], suggesting that any attempts to decrease IT in this population might be beneficial for their mental health and overall quality of life. For instance, research has shown that mentalization moderates the relationship between rejection and internalized transphobia with mental health [107]. Discrimination and internalized stigma are indeed significantly associated with psychological distress in gender minority individuals [108]. Along the same line, given that the “distal” stressors posited by this framework negatively affect the well-being of TGD youth equally, interventions should be based on reducing community-based stigma and discrimination; for instance, and especially, within school-based environments [109]. Research on TGD individuals’ early traumatization also appears as very significant, since research has shown that TGD persons experiencing (or having experienced) traumatic stress tend to report higher rates of mental health issues such as depression, suicidality, and substance use [110], and to be emotionally, physically, and sexually abused more than their cisgender counterparts [111]. Since traumatic experiences, and especially ACEs, have a negative impact on young individuals’ emotion and affect regulation, as well as treatment engagement [112], trauma-informed mental health providers can aid TGD youth to build resilience capacities [113], and ultimately achieve post-traumatic growth [114]. Ultimately, in order to inform treatment procedures with positive therapeutic benefits, it seems necessary to better investigate the complex relationships existing between mental health issues, psycho-social functioning, gender dysphoria, traumatic experience, and developmental settings [62]. 

### 4.5. External and Cultural Factors

Watson and colleagues’ [80] study, which found that experiencing bullying was more common for TGD youth living in states with discriminating or “conversion therapy” laws, where alcohol use and abuse was, however, less common, seems to suggest that state-based laws promoting equality are necessary, but alone, they are insufficient to prevent TGD individuals from using substances. Indeed, a complex array of factors must be taken into account to prevent behavioral problems such as substance use among TGD youth. The high levels of psychopathology reported by TGD individuals have been thought of as being associated with cultural factors such as social ostracism, and with generic risk factors for psychopathology, including family environments [115]. On this basis, family- and community-based environments should pay more attention to the discrimination and victimization of this population.

### 4.6. Internalizing Symptoms and Emotion Regulation

Compared with previous research, including samples of TGD youth who had not socially transitioned [116,117], Durwood and colleagues’ [14] findings related to TGD youth who have socially transitioned seems to suggest different patterns of mental health-related variables (i.e., depression, anxiety, and self-worth) in the latter population, perhaps due to the fact that the children in their study believed they actually were the “opposite” gender, compared with just wishing or preferring to be another gender, which was a significant characteristic of participants in previous studies [118]. In addition, in contrast with previous studies [119,120], Kuper and colleagues [62] found, instead, higher levels of internalizing problems in transgender females rather than in males. Such mixed results and differences in the measures of mental health outcomes, emotion regulation, and highly risky behaviors in TGD youth might be due to several variables involved in the studies, such as the recruitment methods, age of the sample, and the time frame of the measures [58]. 

### 4.7. The Role of Social Support

The negative mental health observed in TGD youth is often accompanied by lower levels of social support compared with cisgender counterparts [121], which does not protect this population from experiencing discrimination, stigma, and prejudice [40]. TGD youth can face discrimination and victimization also within the family they belong to, which can be physically assaultive, disowning their identity, or embracing more subtle behaviors, for instance, using the wrong name or pronoun (i.e., “misgendering”) or preventing them from affirmative care [60]. Accordingly, the capacity and willingness of family members to support TGD youth’s identities seems to be associated with the positive well-being of the entire family [122]. However, protective factors also emerged as substantially significant for TGD youth’s mental health. Even though levels of support tend to be lower for TGD youth than for their cisgender peers, feelings of support by parents, teachers, and peers seems to help these individuals face social stigma and discrimination [90]. However, the fact that poor family functioning seems not to increase the effects of peer problems, and that adequate family functioning does not seem to buffer the effects of peer problems, suggests that both peer and family relationships are independently associated with TGD youth mental health [65]. Accordingly, since problems in either peer or family relationships is associated with increased internalizing symptomatology, it might thus be necessary to work on both dimensions to enhance a TGD youth’s quality of life. Since access to gender-affirming care is only a part of the freedom to live with one’s chosen gender identity, family and peer support can substantially contribute to the overall mental health of young TGD persons, and specifically, children and adolescents, thus helping them to socially transition and affirm their experienced gender both within one’s home and in public spaces [60]. For instance, self-compassion—which is comprised of a system of three interrelated elements (i.e., mindfulness, self-kindness, and common humanity) [123]—seems associated with lower levels of suicidal ideation and behavior among lesbian, gay, bisexual, transgender, and queer (LGBTQ) youth [124]. On this basis, self-compassion has been deemed to be a significant resource to face minority stressors and stigma for transgender individuals [54]. 

### 4.8. Gender Role Discrepancy Stress

Gender role discrepancy strain and discrepancy stress is a form of gender-based stress that stems from the failure to live with one’s assigned gender [125]. For instance, MDS refers to a type of gender role stress stemming from the fear of being perceived to not conform to the assigned gender role [126]. Accordingly, MDS might lead men and boys to engage in aggressive and violent behavior as a means to stereotypically demonstrate masculinity [127]. Accordingly, feminine discrepancy stress seems to be a person- and context-level type of stress that leads women to perceive diminished self-esteem [128]. Given that more than half of TGD youth report ever considering suicide (with almost one in three reporting a suicide attempt), health professionals, school personnel, community organizations, and families should recognize, protect, and provide support to this vulnerable population [58]. For young TGD individuals, affirmative approaches can aid in creating a positive self-narrative, counteracting social stigma, and achieving self-acceptance [111,129]. Clinicians can thus validate these persons’ experiences and help them empower their authenticity by constructing a new personal identity [56]. Ultimately, prevention strategies should not concentrate on a singular etiology [71], but take into account the complex variety of factors influencing the health and well-being of TGD youth.

### 4.9. Sex Trading and Sexual Exploitation

A specific at-risk population comprises TGD youth who trade sex [67]. These individuals can find help in school and clinical contexts, such as mental and sexual health providers, who might use trauma-informed, healing-focused, and affirming approaches to this issue. Spotting trauma in the TGD population might indeed aid in providing care based on an ecological, trauma-informed approach [130], which can support TGD youth to achieve resilience and post-traumatic growth [129]. Overall, more research is needed to disentangle the mutual interactions between mental health issues, traumatic experience such as ACEs, emotional well-being, minority stressors and resilience factors, and gender felt pressure, in that they might have different consequences on the person’s quality of life [56]. In addition, understanding sexual exploitation is of paramount importance and requires a non-judgmental and non-pathologizing approach, in order to deconstruct and challenge the dominant narratives and expectations about gender and sexuality. Clinicians working within therapeutic settings, as well as clinical doctors working in endocrinological clinics, should be aware that decreasing negative emotions in TGD youth can significantly improve the affective dimensions of these individuals. 

Finally, even though most included articles are relatively recent (indeed, some of them have been published after the COVID-19 outbreak), only one addressed the effects of the pandemic on TGD youth’s mental health [70]. This directly points to the need to implement studies on the effects that isolation and social restrictions, which resulted from the governmental measures taken to prevent the spread of the virus in various countries, had not only on the general population, but also, and especially, on young TGD individuals.

### 4.10. Developmental Trajectories of TGD Children and Adolescents

The developmental trajectories of children and adolescents tend to be very open-ended as to their overall sexual identity in all its dimensions (e.g., gender identity, sexual orientation, etc.). Indeed, these individuals undergo rapid changes in terms of physical and psychological development. Therefore, it might be possible that a child who experiences a type of gender diversity at an early stage turns out to be cisgender at a later moment in life, as attested by what scholars usually call “desisters” (i.e., those who cease to identify with a gender other than the one assigned at birth) [131]. Since gender identity in children and adolescents is a developmental task, to experience some type of TGD is not predictive of a particular outcome in terms of gender identity. Specifically, children who demonstrate early TGD will not necessarily follow this trajectory. 

However, the studies addressing gender felt pressure [51] show that the latter seriously limits the exploration of one’s gender identity. Indeed, felt pressure hinders the exploration of one’s nascent and/or developing identity, and represents an obstacle to the individuation of one’s perceived gender. Therefore, health care professionals working with TGD children and adolescents should favor the open and respectful exploration of their gender identity [132], so that unexplored aspects of their personality might not hinder the free development of their structured identity. Ultimately, feeling gender pressure less oppressively can help a person to better define their gender identity. Future research should be aimed at comparing the developmental trajectories of TGD children and adolescents, who have a more rapidly changing identity, and the characteristics of TGD adults, who might have different needs.

Overall, these results do not suggest that all transgender individuals face mental health issues, but that they suffer from additional stressors (minority stressors) compared with cisgender people, and that this might lead to poorer mental health outcomes. The latter do not obviously depend on their gender identity, but precisely on the societal pressure to conform to one’s assigned gender, which exerts pressure on the person’s self-expression. Of note, the included articles tend to stress the negative aspects of the TGD populations’ mental health. Indeed, in order not to convey the idea that the TGD population is powerless and depleted, according to a “damage-centered” approach [133], we intend to highlight the importance of conducting studies that embrace an agency-focused perspective, aimed at empowering this population by analyzing their positive interpersonal functioning [134]. Indeed, what emerges from our work is that the TGD population is able to mobilize resilience factors that are crucial in facing minority stressors. Therefore, the overall aim is to render TGD individuals empowered and not depowered.

## 5. Limitations

Some limitations apply to the present systematic review. First, the literature search was conducted in three scientific databases (i.e., Scopus, PubMed, and Web of Science). It is thus possible that we did not find relevant records because they were not present in these databases and that yet corresponded to our inclusion criteria. We tried to circumvent this limitation by searching in-text relevant citations within the included articles. Second, the systematic review is limited to studies published in English and in peer-reviewed journals. Therefore, records not in English might have been relevant but were excluded from our systematic review. Along the same line, grey literature was excluded, even though records might have been present that were relevant to our search. A further limitation consisted of excluding theoretical and qualitative records that might have added additional insights. As in the first case, we tried to consider them as well in this systematic review, albeit not in the results section. Finally, most studies included in this systematic review had a cross-sectional study design, which prevents the establishment of causal relationships among variables. Future research should, thus, further implement longitudinal studies on this topic.

## 6. Conclusions

Our systematic review highlighted that TGD youth (i.e., children and adolescents), even though often showing high levels of resilience to face minority stressors, tend to suffer from mental health issues (e.g., depression and anxiety) at a greater extent than the cisgender population, mainly due to the gender-based pressure they feel from society to conform to their assigned gender. In order for young TGD individuals to attain a better quality of life, it is necessary that multiple support systems mobilize their resources to meet their specific needs. Resilience factors often comprise relational bonds such as peer-, family- and community-connectedness, which can consistently buffer the negative effects that stigma, trauma, and discrimination have on this population’s mental outcomes. Decreasing negative emotions, also with the help of mental health professionals, can significantly improve self-efficacy and acceptance. Ultimately, affirmative approaches have been shown to aid this population in achieving a better quality of life through the construction of a positive self-concept and freedom from internalized stigma.

## Figures and Tables

**Table 1:** Summary of included articles.

Author(s)	Country	Study Design	Sample Size	[Age Range] Mean (SD)	Types of Respondent	Measures
Becerra-Culqui et al., 2018 [53]	N/A	Cross-sectional	1333 TGD, 26,300 cisgenders	[3–17] N/A	TGD youth	Anxiety disorder, ADHD, ASD, conduct and/or disruptive disorder, depressive disorder, eating disorders
Bluth et al., 2021 [54]	USA and Canada	Non-randomized experimental	41	[13–17] 14.5 (1.49)	TGD adolescents	Self-compassion (17 items from the SCS-Y), student life satisfaction (SLSS), state anxiety (ANX-SF), depression (PHQ-9), interpersonal needs (INQ), resilience (BRS)
Cheung et al., 2020 [55]	Thailand	Cross-sectional	2070	[13–18] N/A	TGD adolescents	Gender role conformity, peer victimization, current health risk behaviors, depressive symptoms
Chodzen et al., 2018 [12]	USA	Cross-sectional	109	[12–18] 15.46 (1.55)	TGD adolescents	Mental health (i.e., Major Depression Disorder and Generalized Anxiety Disorder) (YI-4), appearance congruence (TCS, AC subscale), minority stress and resilience (GMSR)
Clark et al., 2014 [12]	New Zealand	Cross-sectional	8166	N/A	TGD adolescents	Awareness of or disclosure about being transgender, protective factors (i.e., feeling cared about, family relationships), violence and personal safety, mental health (i.e., depression, self-harm, suicide attempts, having drunk alcohol in the past month, having had sex, health care access)
Craig et al., 2020 [56]	USA and Canada	Cross-sectional	3508	[14–18] 16.02 (1.24)	TGD adolescents	ACEs (ACE Scale)
Durwood et al., 2017 [14]	USA and Canada	Cross-sectional	(a) for depression and anxiety measurements: 63 TGD, 63 cisgenders, 38 siblings (b) for self-worth measurements: 116 TGD, 122 cisgenders	(a) [9–14] 10.8 (1.3) (b) [6–14] N/A	TGD youth, caregivers	Internalizing psychopathology (PROMIS scale); self-worth (Global Self-Worth Subscale from the Harter Self-Perception Profile for Children)
Durwood et al., 2021 [57]	USA	Cross-sectional	265	[3–15] 9.41 (2.62)	Caregivers	Family-, peer-, school-, and state-level support, victimization, internalizing symptoms (NIH PROMIS Scales for Anxiety and Depression, parent proxy short forms)
Eisenberg et al., 2017 [58]	USA	Cross-sectional	2168	[14–17] N/A	TGD adolescents	Risk behaviors and experiences (i.e., substance use, sexual behavior, emotional distress, bullying, victimization), emotional distress (i.e., depression, anhedonia) (PHQ-2), family connectedness, teacher-student relationship (SEI), feeling of safety in the community
Hill et al., 2010 [59]	USA and Canada	Cross-sectional	31 TGD, 42 parents (16 mother and father or lesbian couples; 16 one parent)	[4–17.5] 8.0	Caregivers	Emotional and maladaptive behaviors (CBCL), extent of child cross-gendering (GIQ), anti-transgender attitudes (GTS)
Katz-Wise et al., 2018 [60]	USA	Cross-sectional	96: (a) 33 TGD youth (b) 48 cisgender caregivers (c) 15 cisgender siblings	(a) [13–17] 15.18 (1.24) (b) [37–69] 50.33 (6.70) (c) [14–24] 17.93 (3.28)	TGD adolescents, cisgender caregivers, cisgender siblings of TGD adolescents	Family communication (8-item subscale from FACES IV), family satisfaction (10-item subscale from FACES IV), suicidality (YRBSS), self-harm, depression (CES-D Short Form), anxiety (SCAS), self-esteem (RSES), resiliency (READ)
Kolbuck et al., 2019 [61]	USA	Cross-sectional	71	[3–11] 7.79 (2.08)	Caregivers	Gender nonconformity (GIQC), parenting stress (PSI-SF), parental support (PSGV), psychological functioning (CSI and ECI)
Kuper et al., 2019 [62]	USA	Cross-sectional	396: 149 TGD youth; 137 mothers; 110 fathers	[12–18] 15.3 (1.52)	Adolescents, caregivers	CBCL (completed by parents/guardians), YSR and BIS (completed by adolescents)
Lowry et al., 2018 [9]	USA	Cross-sectional	6082	[14–18] N/A	TGD adolescents	Gender expression, sexual identity, substance use and mental distress (YRBS)
Mahfouda et al., 2019 [63]	Australia	Cross-sectional	104 TGD	14.62 (1.72)	Children, caregivers	Autistic traits (SRS-2), behavioral and emotional difficulties (YSR), health-related quality of life and adaptive functioning (Pediatric Quality of Life Inventory)
Moyer et al., 2018 [64]	USA	Cross-sectional	79	[11–18] 15.6 (1.8)	Medical doctor	Depression (PHQ-9), anxiety (GAD-7)
Munroe et al., 2020 [65]	USA	Longitudinal	45 TGD children; 45 caregivers	[6–12] 8.5 (1.5)	Caregivers	Emotional and maladaptive behaviors (CBCL), caregiver-rated family functioning (Family Assessment Device), peer problems (peer relations subscale of the CBCL)
Nahata et al., 2017 [66]	USA	Retrospective cohort	79	[9–18] 15	TGD youth	Insurance coverage, mental health diagnosis (i.e., depression, anxiety, PTSD, eating disorders, ASD, bipolar disorder), self-injurious behavior (i.e., suicidal ideation, self-harm, suicidal attempts), school victimization
Nic Rider et al., 2022 [67]	N/A	Cross-sectional	67,806 (1024 TGD; 66,782 cisgenders)	[14–17] N/A	TGD youth, cisgender individuals	Sex trading, mental health, teacher-student relationship (4 items from SEI), feeling of safety at school
Olson et al., 2016 [68]	USA	Cross-sectional	73 TGD; 49 siblings; 73 cisgender controls	[3–12] 7.7 (2.2)	Caregivers	Symptoms of anxiety and depression (PROMIS—Parental Proxy short forms for anxiety and depression)
Parodi et al., 2022 [69]	USA	Cross-sectional	252	[14–18] 16.07 (1.01)	TGD adolescents	Anxiety (GAD-7), depression (PHQ-2), non-suicidal self-injury (Youth Risk Behavior Survey), PTSD (PC-PTSD-5), school connectedness (PSSM), presence of GSAs, nondiscrimination laws in the state of residence
Perl et al., 2021 [70]	Israel	Cross-sectional	76 (18 TGD, 58 cisgender)	[9–18] N/A	TGD youth	Coronavirus health impact (CRISIS-V0.2), emotion regulation (ERQ)
Reidy et al., 2018 [71]	USA	Cross-sectional	592	[11–16] 13.1 (1.6)	TGD youth	GRD and masculine discrepancy stress (MDSS), trauma (Child PTSD Symptom Scale), neighborhood disorganization (RYDS), ACEs, psychosocial adjustment, substance use, sexual behavior, mood disorder symptom (K6), hopelessness (HSC), violence (NYS)
Reisner et al., 2015 [26]	USA	Cross-sectional	5907	[13–18] N/A	TGD adolescents	Gender minority identity, past-12-month ever and regular substance use, past-12-month bullying experiences
Russell et al., 2022 [72]	USA	Cross-sectional	58	[9,10] 10.03 (0.62)	Caregivers	Emotional and maladaptive behaviors (CBCL)
Shumer et al., 2015 [73]	USA	Cross-sectional	2128 youth, 2876 caregivers	[9–14] N/A	Youth, mothers	Social functioning, reciprocal social interaction, restrictive or stereotypical behaviors associated with ASD (SRS)
Stewart et al., 2021 [74]	USA and Canada	Cross-sectional	94,804	[4–18] 12.1 (3.72)	Medical doctor	Youth’s mental health needs and risks (interrail ChYMH), child’s functioning and aids (interRAI ChYMH Screener (ChYMH-S), child’s functioning (interRAI ChYMH Screener)
Turban et al., 2021 [75]	USA	Cross-sectional	9711	[18–65+] 34.8 (13.9)	TGD adults	Mental health, age recalled for social transition
Vance et al., 2021 [76]	USA	Cross-sectional	19,780: 252 Black and Latinx TGD individuals, 104 White TGD individuals, 19,424 Black and Latinx cisgender individuals	[14,15] N/A	TGD adolescents, cisgenders	Mental health (depressive symptomatology, suicidal ideation), school-based victimization, harassment, school connectedness
VanderLaan et al., 2017 [77]	N/A	Cross-sectional	360 AMAB	[N/A] 6.86 (2.31)	TGD children	Separation anxiety (SAI), gender non-conformity (GIIC, GIQC), CBCL
Van der Miesen et al., 2018 [78]	Canada	Cross sectional	1719	[6–12] N/A	Caregivers	Extent of childhood cross-gendering (GIQC), emotional and maladaptive behaviors (CBCL)
Wang et al., 2020 [79]	China	Cross-sectional	12,108: 2111 TGD, 9997 cisgenders	[N/A] 15.8 (1.0)	TGD and cisgender adolescents	Physical health (SFSI, item 1), depressive symptoms (PHQ-9), anxiety symptoms (GAD-7), sleep quality (CPSQI), bullying, self-harm and suicidal ideation
Watson et al., 2021 [80]	USA	Cross-sectional	8831	[13–17] 15.59 (1.27)	TGD adolescents	LGBTQ equity index, substance use (i.e., recent alcohol use, binge drinking, cigarette use), bias-based bullying

Note: TGD = transgender or gender diverse; ADHD = attention deficit and/or hyperactivity disorder; ASD = autism spectrum disorder; ACEs = adverse childhood experiences; PTSD = post-traumatic stress disorder; GSAs = gay-straight alliances or gender-sexuality alliances; GRD = gender role discrepancy.

**Table 2:** Quality ratings of selected studies.

Author(s)	1a	2a	3a	4a	5a	6a	7a	8a	9a	10a	11a	12a	13a	14a	Overall Rating
Becerra-Culqui et al., 2018 [53]	Yes	Yes	N.R.	Yes	No	No	No	N.A	Yes	N.A.	Yes	N.A.	N.A.	Yes	Good
Cheung et al., 2020 [55]	Yes	Yes	Yes	Yes	No	No	No	Yes	No	No	Yes	N.A.	N.A.	Yes	Good
Chodzen et al., 2018 [18]	Yes	Yes	N.R.	Yes	No	No	No	Yes	Yes	No	Yes	N.A.	N.A.	Yes	Good
Clark et al., 2014 [12]	Yes	Yes	Yes	Yes	No	No	No	Yes	No	No	Yes	N.A.	N.A.	Yes	Good
Craig et al., 2020 [56]	Yes	Yes	Yes	Yes	No	No	No	N.A	Yes	N.A.	Yes	N.A.	N.A.	N.A.	Good
Durwood et al., 2017 [14]	Yes	Yes	N.R.	Yes	No	No	No	N.A	Yes	N.A.	Yes	N.A.	N.A.	N.A.	Good
Durwood et al., 2021 [57]	Yes	Yes	N.R.	Yes	No	No	No	Yes	Yes	No	Yes	N.A.	N.A.	Yes	Good
Eisenberg et al., 2017 [58]	Yes	Yes	N.R.	Yes	No	No	No	Yes	Yes	No	Yes	N.A.	N.A.	N.A.	Good
Hill et al., 2010 [59]	Yes	Yes	N.R.	Yes	No	No	No	Yes	Yes	No	Yes	N.A.	N.A.	No	Fair
Katz-Wise et al., 2018 [60]	Yes	Yes	N.R.	Yes	No	No	No	Yes	Yes	No	Yes	N.A.	N.A.	Yes	Good
Kolbuck et al., 2019 [61]	Yes	Yes	N.R.	Yes	No	No	No	Yes	Yes	No	Yes	N.A.	N.A.	Yes	Good
Kuper et al., 2019 [62]	Yes	Yes	Yes	Yes	Yes	No	No	Yes	Yes	No	Yes	N.A.	N.A.	Yes	Good
Lowry et al., 2018 [9]	Yes	Yes	Yes	Yes	Yes	No	No	Yes	Yes	No	Yes	N.A.	N.A.	Yes	Good
Mahfouda et al., 2019 [63]	Yes	Yes	Yes	Yes	No	No	No	Yes	Yes	No	Yes	N.A.	N.A.	Yes	Good
Moyer et al., 2018 [64]	Yes	Yes	N.R.	Yes	No	No	No	Yes	Yes	No	Yes	N.A.	N.A.	N.A.	Fair
Munroe et al., 2020 [65]	Yes	Yes	N.R.	Yes	No	Yes	Yes	Yes	Yes	Yes	Yes	N.A.	N.A.	No	Good
Nahata et al., 2017 [66]	Yes	Yes	N.R.	Yes	No	No	No	N.A.	Yes	No	No	N.A.	N.A.	N.A.	Fair
Nic Rider et al., 2022 [67]	Yes	Yes	N.R.	Yes	No	No	No	Yes	Yes	No	Yes	N.A.	N.A.	N.A.	Good
Olson et al., 2016 [68]	Yes	Yes	N.R.	Yes	No	No	No	N.A.	Yes	No	Yes	N.A.	N.A.	N.A.	Good
Parodi et al., 2022 [69]	Yes	Yes	N.R.	Yes	No	No	No	Yes	Yes	No	Yes	N.A.	N.A.	Yes	Good
Perl et al., 2021 [70]	Yes	Yes	N.R.	Yes	No	No	No	Yes	Yes	No	Yes	N.A.	N.A.	No	Fair
Reidy et al., 2018 [71]	Yes	Yes	N.R.	Yes	No	No	No	Yes	Yes	No	Yes	N.A.	N.A.	Yes	Good
Reisner et al., 2015 [26]	Yes	Yes	Yes	Yes	No	No	No	N.A.	Yes	No	Yes	N.A.	N.A.	Yes	Good
Russell et al., 2022 [72]	Yes	Yes	N.R.	Yes	No	No	No	Yes	Yes	No	Yes	N.A.	N.A.	Yes	Good
Shumer et al., 2015 [73]	Yes	Yes	N.R.	Yes	No	No	No	Yes	Yes	No	Yes	N.A.	N.A.	Yes	Good
Stewart et al., 2021 [74]	Yes	Yes	N.R.	Yes	No	No	No	N.A.	Yes	No	Yes	N.A.	N.A.	Yes	Fair
Turban et al., 2021 [75]	Yes	Yes	N.R.	Yes	No	No	No	Yes	Yes	No	Yes	N.A.	N.A.	Yes	Good
Vance et al., 2021 [76]	Yes	Yes	N.R.	Yes	No	No	No	Yes	Yes	No	Yes	N.A.	N.A.	Yes	Good
VanderLaan et al., 2017 [77]	Yes	Yes	N.R.	Yes	No	No	No	Yes	Yes	No	Yes	N.A.	N.A.	Yes	Good
Van der Miesen et al., 2018 [78]	Yes	Yes	N.R.	Yes	No	No	No	Yes	Yes	No	Yes	N.A.	N.A.	Yes	Good
Wang et al., 2020 [79]	Yes	Yes	Yes	Yes	No	No	No	Yes	Yes	No	Yes	N.A.	N.A.	Yes	Good
Watson et al., 2021 [80]	Yes	Yes	Yes	Yes	No	No	No	Yes	Yes	No	No	N.A.	N.A.	Yes	Good
	1b	2b	3b	4b	5b	6b	7b	8b	9b	10b	11b	12b	−	−	Overall rating
Bluth et al., 2021 [54]	Yes	Yes	Yes	Yes	No	Yes	Yes	No	Yes	Yes	Yes	Yes	−	−	Good

Notes. 1a = Was the research question or objective in this paper clearly stated?; 2a = Was the study population clearly specified and defined?; 3a = Was the participation rate of eligible persons at least 50%?; 4a = Were all the subjects selected or recruited from the same or similar populations (including the same time period)? Were inclusion and exclusion criteria for being in the study prespecified and applied uniformly to all participants?; 5a = Was a sample size justification, power description, or variance and effect estimates provided?; 6a = For the analyses in this paper, were the exposure(s) of interest measured prior to the outcome(s) being measured?; 7a = Was the timeframe sufficient so that one could reasonably expect to see an association between exposure and outcome if it existed?; 8a = For exposures that can vary in amount or level, did the study examine different levels of the exposure as related to the outcome (e.g., categories of exposure, or exposure measured as a continuous variable)?; 9a = Were the exposure measures (independent variables) clearly defined, valid, reliable, and implemented consistently across all study participants?; 10a = Was the exposure(s) assessed more than once over time?; 11a = Were the outcome measures (dependent variables) clearly defined, valid, reliable, and implemented consistently across all study participants?; 12a = Were the outcome assessors blinded to the exposure status of the participants?; 13a = Was the loss to follow-up after the baseline 20% or less?; 14a = Were key potential confounding variables measured and adjusted statistically for their impact on the relationship between exposure(s) and outcome(s)?; 1b = Was the study question or objective clearly stated?; 2b = Were the eligibility or selection criteria for the study population prespecified and clearly described?; 3b = Were the participants in the study representative of those who would be eligible for the test or service or intervention in the general or clinical population of interest?; 4b = Were all eligible participants that met the prespecified entry criteria enrolled?; 5b = Was the sample size sufficiently large to provide confidence in the findings?; 6b = Was the test or service or intervention clearly described and delivered consistently across the study population?; 7b = Were the outcome measures prespecified, clearly defined, valid, reliable, and assessed consistently across all study participants?; 8b = Were the people assessing the outcomes blinded to the participants’ exposures or interventions?; 9b = Was the loss to follow-up after the baseline 20% or less? Were those lost to follow up accounted for in the analysis?; 10b = Did the statistical methods examine changes in the outcome measures from before to after the intervention? Were statistical tests performed that provided *p* values for the pre-to-post changes?; 11b = Were outcome measures of interest taken multiple times before the intervention and multiple times after the intervention (i.e., did they use an interrupted time-series design)?; 12b = If the intervention was conducted at a group level (e.g., a whole hospital, a community, etc.), did the statistical analysis take into account the use of individual-level data to determine the effects at the group level? CD: cannot determine; NA: not applicable; NR: not reported.

## Data Availability

Not applicable.

## List of Abbreviations

AC	appearance congruence
ACEs	adverse childhood experiences
ADHD	attention deficit and/or hyperactivity disorder
ASD	autism spectrum disorder
CBCL	child behavior checklist
CD	conduct disorder
DSM	diagnostic and statistical manual of mental disorders
GAD	generalized anxiety disorder
GRD	gender role discrepancy
GSAs	gay-straight alliances or gender-sexuality alliances
IT	internalized transphobia
LGBT(Q)	lesbian, gay, bisexual, transgender, (queer)
MDD	major depressive disorder
MDS	masculine discrepancy stress
MST	minority stress theory
PTSD	post-traumatic stress disorder
TGD	transgender and gender diverse
YSR	Youth Self Report
